# Ocean acidification has little effect on the biochemical composition of the coccolithophore *Emiliania huxleyi*

**DOI:** 10.1371/journal.pone.0218564

**Published:** 2019-07-10

**Authors:** Elena Heidenreich, Robin Wördenweber, Frank Kirschhöfer, Michael Nusser, Frank Friedrich, Kirsten Fahl, Olaf Kruse, Björn Rost, Matthias Franzreb, Gerald Brenner-Weiß, Sebastian Rokitta

**Affiliations:** 1 Analytical Biochemistry, Department of Bioengineering and Biosystems, Institute of Functional Interfaces, Karlsruhe Institute of Technology, Eggenstein-Leopoldshafen, Germany; 2 Algae Biotechnology & Bioenergy, Department of Biology, Center for Biotechnology (CeBiTec), Bielefeld University, Bielefeld, Germany; 3 Competence Center for Material Moisture (CMM), Karlsruhe Institute for Technology, Hermann-von-Helmholtz-Platz 1, Eggenstein-Leopoldshafen, Germany; 4 Marine Geology and Paleontology, Alfred-Wegener-Institute, Helmholtz-Centre for Polar and Marine Research, Bremerhaven, Germany; 5 Marine Biogeosciences, Alfred-Wegener-Institute, Helmholtz-Centre for Polar and Marine Research, Bremerhaven, Germany; 6 University of Bremen, Bremen, Germany; Stazione Zoologica Anton Dohrn, ITALY

## Abstract

Owing to the hierarchical organization of biology, from genomes over transcriptomes and proteomes down to metabolomes, there is continuous debate about the extent to which data and interpretations derived from one level, e.g. the transcriptome, are in agreement with other levels, e.g. the metabolome. Here, we tested the effect of ocean acidification (OA; 400 vs. 1000 μatm CO_2_) and its modulation by light intensity (50 vs. 300 μmol photons m^-2^ s^-1^) on the biomass composition (represented by 75 key metabolites) of diploid and haploid life-cycle stages of the coccolithophore *Emiliania huxleyi* (RCC1216 and RCC1217) and compared these data with interpretations from previous physiological and gene expression screenings. The metabolite patterns showed minor responses to OA in both life-cycle stages. Whereas previous gene expression analyses suggested that the observed increased biomass buildup derived from lipid and carbohydrate storage, this dataset suggests that OA slightly increases overall biomass of cells, but does not significantly alter their metabolite composition. Generally, light was shown to be a more dominant driver of metabolite composition than OA, increasing the relative abundances of amino acids, mannitol and storage lipids, and shifting pigment contents to accommodate increased irradiance levels. The diploid stage was shown to contain vastly more osmolytes and mannitol than the haploid stage, which in turn had a higher relative content of amino acids, especially aromatic ones. Besides the differences between the investigated cell types and the general effects on biomass buildup, our analyses indicate that OA imposes only negligible effects on *E*. *huxleyi*´s biomass composition.

## Introduction

The globally distributed coccolithophore *Emiliania huxleyi* is an intensively investigated microalga, especially because of its dual role in biogeochemistry. Besides being photosynthetic phytoplankton that contributes to the so-called ‘organic carbon pump’ of the oceans, it is able to produce elaborately shaped CaCO_3_ shells. This process of calcification alters the surrounding sea-water chemistry, driving the so-called oceanic ‘inorganic carbon pump’ [1]. Also, the heavy calcite material aggregates with various other marine particles, and enhances their export to depth. In this way, marine calcifiers strongly influence the relative strengths of the oceanic carbon pumps [2].

In recent years, the global change phenomenon of ‘Ocean Acidification’ (OA) has stimulated extensive research on calcifying organisms. OA describes the consequences of the uptake of anthropogenic CO_2_ by the surface ocean [3], i.e., a drop in pH and thus lowered chemical saturation states for carbonate minerals, putting marine calcifiers and the inorganic carbon pump at stake [4]. In the course of intensified research over the past decades, *E*. *huxleyi* has become a model organism for calcifying marine microalgae, not only because it is globally distributed with many ecotypes from different ocean provinces [5], or because many of these ecotypes were shown to be highly responsive to ocean acidification [6–8], but also because its genome has been sequenced [5], enabling the exploitation of wider ‘omics’ approaches. Especially RNA- and protein-based approaches have since then delivered insights into the ecophysiology of the life-cycle stages of *E*. *huxleyi* [9,10] and the responses of this microalga towards a set of diverse environmental triggers and stimuli, like prolonged darkness [11], increased light intensity [12], nutrient starvation [13–17] and also OA [18–23].

Previously, Rokitta and Rost [20] investigated on a physiological basis the responses of *E*. *huxleyi* strain RCC1216 and its haploid, non-calcifying life-cycle stage (strain RCC1217) towards OA and their modulation by light energization. It was interpreted that OA stimulated biomass production by increased photosynthesis and decreased production of calcite. The relative responses towards OA were found to be weaker under high light despite higher overall rates. In addition, the higher biomass production was sustained by less pigment per biomass, and it was concluded that under OA, cells operate at higher energy-efficiency.

In a subsequent study, transcriptomic analyses from these experiments were conducted using microarray-based transcriptome screenings, in which the expression of ~10.000 genes was assessed. Based on the gene expression patterns, it was interpreted that the increased biomass production likely originates from an intensified lipid metabolism, because especially genes for buildup and oxidation of lipids were found to be significantly regulated, with more synthetic machinery rather than degradative machinery being regulated. Also, genes of the oxidative branch of the pentose-phosphate pathway were found upregulated, which was taken as a supportive indication for increased lipid synthesis, as this pathway creates cytoplasmic reduction equivalents necessary for lipid synthesis [19]. Further investigations on OA-effects in *E*. *huxleyi* targeting the proteome level were complicated by methodological biases. For instance, the shotgun-proteomics techniques available at date yielded merely ~100 protein identifications, and expression studies concerning the effects of OA yielded <10 significantly regulated proteins that were related to protein translation and DNA maintenance [24,25]. Despite the possibility that there is indeed little regulation on the proteome level, the low number of obtained significant features did not permit a ground-truthing of the previously acquired transcriptomic data and the deductions made about the cellular effects of OA.

In addition, there is ongoing debate about consistency of interpretations derived from the newly emerging and highly diverse ‘omics’ techniques that investigate the genomes, transcriptomes, proteomes, and metabolomes of organisms [26]. While the sequencing of genomes delivers insights into the biochemical potential and theoretical abilities of organisms, investigations on the transcriptomes shed light on the expressed genetic machinery that is principally available to the cell in certain environmental settings. However, not all RNAs are equally translated and long-lived, so that there can be considerable differences to the proteome. Lastly, the posttranslational short-term regulation of enzymatic machinery, substrate-regulation, as well as cellular signaling determine the instantaneous biochemical fluxes that ultimately define the metabolic phenotype of living cells. Thus, depending on the respective level of biological organization that is looked at, researchers may draw biased conclusions toward the reactions of cells to specific environmental stimuli and experimental treatments [12,27,28].

To shed further light on the connections between these different abstraction layers of biological organization, we have here conducted metabolite screenings, testing the effects of OA on the metabolic composition of the diploid and haploid life-cycle stages of *E*. *huxleyi*. Because light was shown to strongly modulate the OA responses [19], we acclimated cells to a matrix of contemporary and future pCO_2_ (400 vs. 1000 μatm) under limiting and saturating light intensities (50 vs. 300 μmol photons m^-2^ s^-1^), thereby reproducing the experimental setup of Rokitta and Rost [20]. A main focus was the question whether and to which degree metabolite patterns correlate with the transcriptomic responses and confirm the deductions made based upon them.

## Materials and methods

### Experimental setup

Diploid (2N) and haploid (1N) life-cycle stages of *E*. *huxleyi* (RCC 1216, calcifying; RCC 1217, non-calcifying) were grown in sterile filtered (Sartobran 200 capsule filter, Sartorius, Göttingen, Germany) North Sea seawater medium, enriched with vitamins and trace metals according to the recipe for F/2 medium [29]. Medium was supplemented with nitrate (NO_3_^-^, 100 μM) and phosphate (PO_4_^2-^, 6 μM). Cultures were grown at 15°C in a 16:8 h light/dark cycle under irradiances of 50 or 300 μmol m^-2^ s^-1^ (low-light, LL vs. high-light, HL) provided by daylight lamps (FQ 54W/965HO, OSRAM, Munich, Germany). All media were pre-aerated (~150 mL min^-1^) with air-mixtures of the desired pCO_2_. Air-mixtures were produced by mixing CO_2_-free air with pure CO_2_ (Air Liquide Deutschland, Düsseldorf, Germany) using a mass flow controller system (CGM 2000 MCZ Umwelttechnik, Bad Nauheim, Germany). The pCO_2_ was regularly controlled with a non-dispersive infrared analyzer system (LI6252, LI-COR Biosciences, Bad Homburg, Germany). Precultures were grown in pCO_2_ adjusted media for at least 7 days in exponential growth and were then used as inoculum for the main incubations. Three biological replicates of each life-cycle stage were inoculated for each setup at densities of ~2000 cells mL^-1^ in 2.5 L Schott glass bottles without headspace, which were then placed on a roller table (~15 rpm). The cell numbers were determined in 2–3 day intervals with a Multi-Sizer 3 particle counter (Beckman Coulter), and specific growth rates were derived [30].

Carbonate chemistry was determined in cell-free media prior to acclimations (t_0_), as well as directly prior to cell harvest for metabolome analyses (t_fin_). Calculations of the carbonate system (using the CO2SYS program, [31]) were based on pH and concentrations of dissolved inorganic carbon (DIC) (S1 Table). The pH was measured with a portable pH meter (826 pH mobile, Metrohm, Filderstadt, Germany) with an electrode containing an integrated temperature sensor (Aquatrode Plus with Pt 1000, measurement reproducibility ± 0.01 pH units) and were corrected for temperature and daily offsets by independent measurement of a TRIS Buffer [32]. Three-point-calibration of the pH meter was performed every second day with NIST buffer standards (AppliChem, Darmstadt, Germany). Further input parameters for the calculation of the carbonate system were temperature (15°C), salinity (32), and pressure (1 dbar), as well as phosphate (6 μmol kg^-1^) and silicate (7 μmol kg^-1^) concentrations. Calculations were based on equilibrium constants by Mehrbach et al. [33], refit by Dickson and Millero [34] and on dissociation constants for sulfuric acid provided by Dickson [35].

### Harvest

Cells were harvested during exponential growth phase by gentle vacuum filtration (800 mbar suction pressure) onto polycarbonate filters (Isopore TSTP membranes, 3.0 μm pore size, Millipore, Billerica, Massachusetts). For the analyses of pigments, lipids and all other metabolites, culture volumes drawn onto the filters were 300, 300, and 2000 mL, respectively. Filters were snap-frozen in liquid N_2_ and stored at -80°C. Samples were then freeze-dried before analyses to rule out degradation during transport.

### Electron microscopy

5 mL of diploid cell culture were filtered onto 25 mm polycarbonate filters (Nuclepore Track-Etched Membranes, 2 μm pore size, Whatman, Maidstone, UK) and were dried and stored at room temperature. Samples were stuck on holders and sputtered with a thin conductive layer (5 nm Au/Pd 80/20) using a MED-020 sputter coater (Bal-Tec, Balzers, Liechtenstein). Electron microscopic imaging was performed with an XL-30 field emission environmental scanning electron microscope (ESEM; Philips, Eindhoven, Netherlands).

### Metabolite analyses

Most analysis methods and the chosen instruments applied here have been described in detail in Wördenweber et al. [36]. Therefore, these methods will only be explained briefly here. All solvents and chemicals used were LC-MS grade. Small metabolites were extracted with a mixture of methanol:water (80:20, v/v). From this extract, amino acids, small organic acids, mannitol and osmolytes were quantified via LC-MS/MS. Pigments were extracted with 90% (v/v) acetone and analysis was performed via UV-VIS spectroscopy after HPLC separation. For lipid extraction, cells were first removed from the filters with methanol. Subsequently, lipids were extracted with a mixture of methanol, chloroform and water (1:2:0.75, v/v), and then separated into neutral and polar lipid fractions via silica gel column chromatography with pure chloroform and methanol, respectively. Using a part of the neutral lipid fraction, alkenones were purified (first elution of unwanted lipids with dichloromethane/hexane (1:1, v/v), then pure dichloromethane to elute alkenones). Long-chained alkenones were analysed using GC-FID. Analysis of fatty acid methyl-esters (FAMEs) was performed via GC-MS. Triacylglyceride (TAG) analysis was carried out using charged aerosol detector HPLC-CAD. Numerous TAGs (named TAG 1–10) could not be identified using the commercially available TAG standards that contain three identical chain lengths. Thus, they probably consisted of non-identical chain lengths for which standards are not available. The unidentified TAGs were labeled according to Wördenweber et al. [36]. One additional TAG was detected, which eluted between TAG 9 and TAG 10, which was therefore called TAG 9a, for better comparison with the aforementioned study. Mannitol was quantified with a modified method of Kubica et al. [37]. An UHPLC system (ExionLC AD UHPLC, ABSciex, Darmstadt, Germany) was used for separation, coupled with a triple quadrupole MS/MS (API4000, ABSciex, Darmstadt, Germany). Standard substances and samples were diluted with a mixture of acetonitrile/water (50:50, v/v). The substances were separated on an Amide Column (ACQUITY UPLC BEH), 130Å, 1.7 μm, 3 mm x 150 mm (Waters, Eschborn, Germany) with 0.7 mL min^-1^ flowrate and 1 μL injection volume. The column oven was set to 35°C. Eluents were acetonitrile with 5% water (A) and 0.05% acetic acid in water (B). The gradient was run from 25% B for 2 min to 60% B within 0.5 min and held for 1.3 min. Eluent B was set back to 25% within 0.2 min and the column was re-equilibrated for 3 min. D-Mannitol standard substance (BioUltra, ≥99.0%, Sigma) was used for external calibration.

The ‘fold-changes’ of all metabolites were calculated and represented in a heatmap. When a metabolite was detected in one treatment but not at all in the other, a tendency of fold-change was added manually with the value of 1.5 or -1.5 respectively. For clarity, these metabolites have been marked with a slash ‘/’.

### Statistical analysis

Results of metabolite analysis are reported either normalized per cell or normalized to biomass, using the particulate organic carbon (POC) quotas published earlier for this strain of microalgae [20]. All data was calculated from three biological replicates for each treatment. A two-tailed Student’s *t*-test was performed, and statistically significant differences were indicated by asterisks, representing *p*-values ≤0.05 (*) and ≤0.01 (**). All results are shown as fold-changes of mean values, with standard deviation (±SD).

Statistically significant changes in metabolite abundances between the different treatments and life-cycle stages, as determined via Student’s *t*-test (*p*-value ≤0.05), are indicated by asterisks. Results were then correlated with previous transcriptomic and proteomic findings and interpretations.

## Results and discussion

We investigated the effects of OA on the metabolic composition of calcifying diploid and non-calcifying haploid *E*. *huxleyi* life-cycle stages and their modulation by light, i.e. cellular energization. Results are first reported for each life-cycle stage with regard to OA responses and associated modulation by energy availability. Then, the influence of light intensity will be discussed separately, followed by differences in the life-cycle stages.

### Growth, carbonate chemistry and SEM analyses

**Fig 1:**
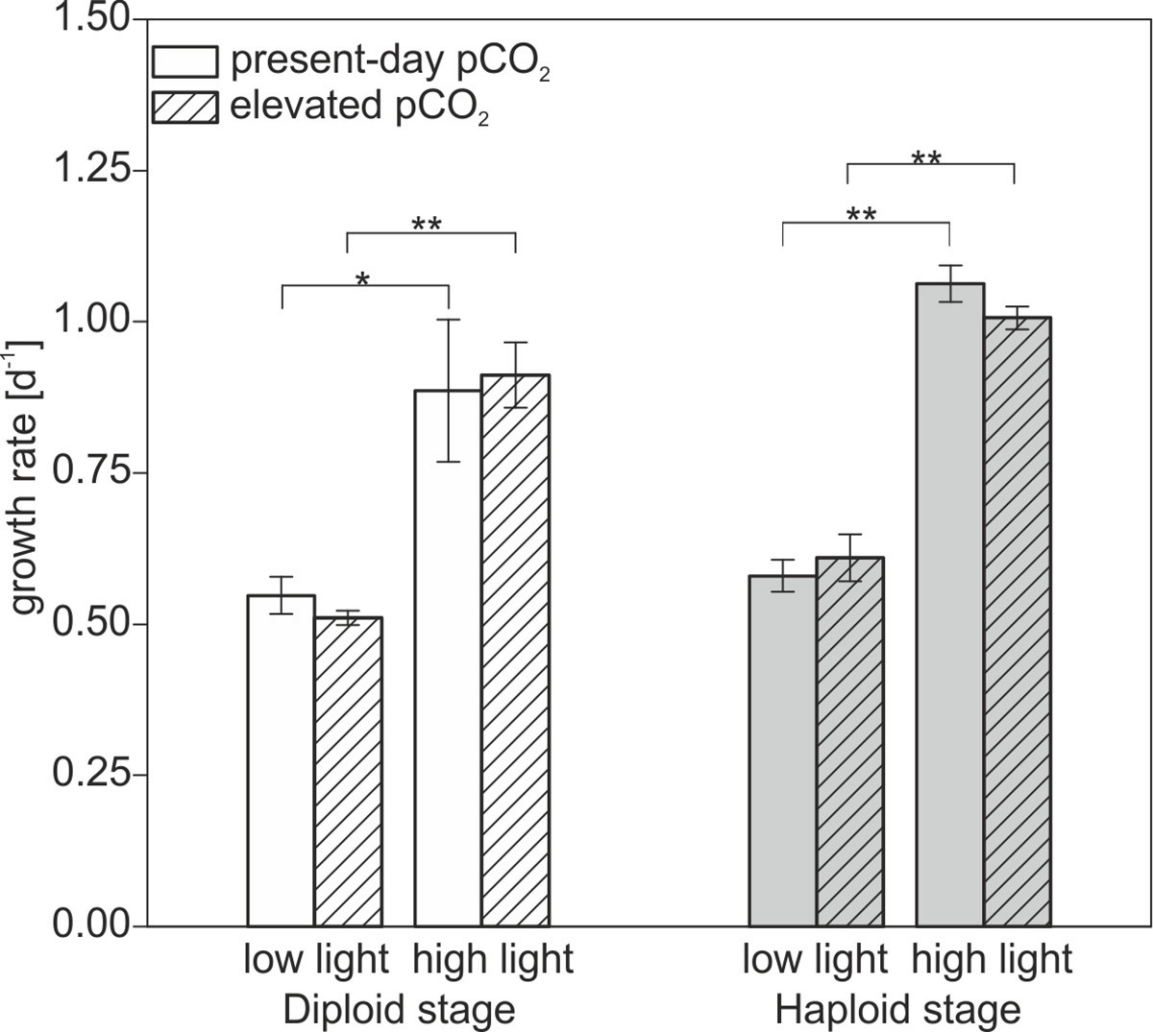
Growth rates. Diploid and haploid *E*. *huxleyi* life-cycle stages under present-day pCO_2_ (empty bars, 400 μatm) and elevated pCO_2_ (hatched bars, 1000 μatm) as well as low and high light intensities. Error bars denote 1 SD (n = 3). Significances were determined via Student’s *t*-test, * = *p*<0.05, ** = *p*<0.01.

In both life-cycle stages, growth rates were not affected by OA. High light, in turn, caused significantly higher growth rates, irrespective of pCO_2_ (Fig 1). The cell concentrations were intentionally kept low to keep the carbonate chemistry quasi-constant throughout the experiment. As a consequence, the concentration of dissolved inorganic carbon (DIC) decreased only slightly in all cultures due to the buildup of biomass and calcite (in diploid cells). Over the course of the cultivation, the total alkalinity (TA) of the haploid cultures stayed more or less stable but decreased in the diploid cultures because of calcification [38]. The detailed carbonate system data for the beginning and the end of the cultivation can be found in the supplementary material (S1 Table).

**Fig 2:**
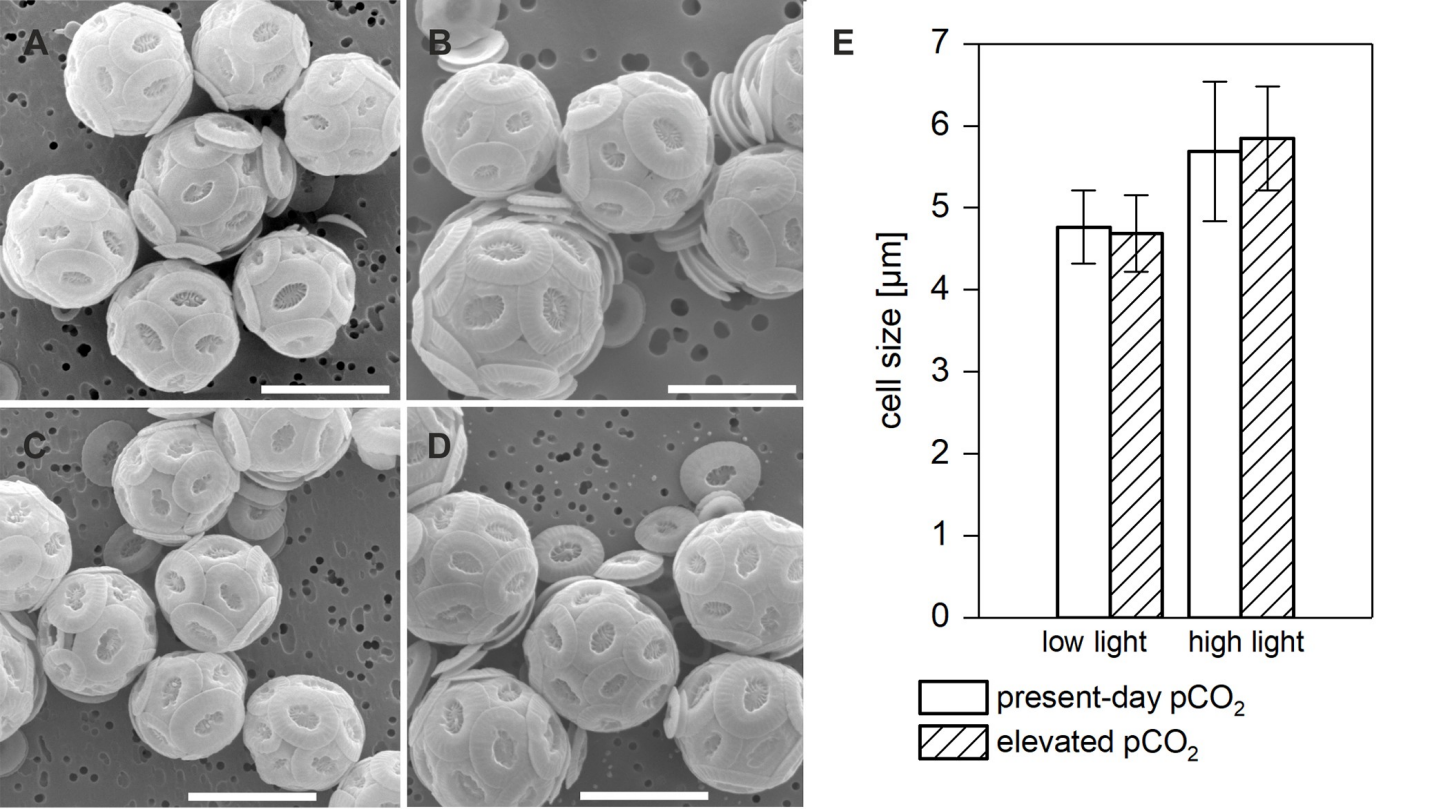
SEM images of *E*. *huxleyi* (RCC1216) coccospheres. A) Low light, 400 μatm pCO_2_ B) High light, 400 μatm pCO_2_ C) Low light, 1000 μatm pCO_2_ D) High light, 1000 μatm pCO_2_. All scalebars 5 μm. Estimates on size diameter are based on metric assessments (n = 33–40 cells). E) Average cell diameter of diploid cells under the different treatments.

Visual inspections (Fig 2A–2D) with a scanning electron microscope and metric assessments of 33–40 coccospheres of diploid cells (Fig 2E) from every treatment showed that pCO_2_ did not have significant effects on the overall size of the cells including the surrounding coccosphere, independent of the applied light level. No negative effects on coccolith morphology through OA were evident. Diploid cells grown under high light were slightly bigger than those grown under lower light irrespective of pCO_2_.

This data shows that the diploid life-cycle stage experienced neither alterations in growth rates nor in coccolith morphology and coccosphere diameter (Figs 1 and 2). These results are in line with previous findings that OA is not a strong determinant of growth rate in this strain of *E*. *huxleyi* [18,20].The resilience of the calcification process against OA-induced malformations has also been observed previously in this particular strain [39]. The unchanged outer diameter of coccospheres might be explained by previous assessments of OA effects on the quotas of particulate organic and inorganic carbon (POC, PIC), i.e. biomass and calcite contents. It was shown that OA affects the partitioning of carbon between these carbon sinks in such way that POC production is stimulated, whereas PIC production is hampered to the same degree by OA [7,20]. As a result, the content of total particulate carbon, i.e., the sum of the two, was unaffected by OA irrespective of irradiance levels and might explain stable coccosphere sizes.

In response to high light, cells of the diploid life-cycle stage generally grew faster and also bigger than under low light and possessed more coccoliths per cell (Figs 1 and 2). These responses were independent of the applied CO_2_ acclimation and coherent with increased growth rates as well as higher production rates in PIC and POC observed earlier under high light [20,39,40].

The growth rate of the haploid stage was not significantly influenced by OA (Fig 1). Other studies have shown significant negative effects of OA on the growth rate of haploid *E*. *huxleyi* [20,41], however these were numerically mostly negligible. Opposite effects were observed only for haploid life-cycle stages of another *E*. *huxleyi* strain where the growth rate increased with higher pCO_2_ [42]. In fact, based on the negligible effects of OA on growth, elemental quotas and production rates, it was concluded that the haploid stage of this *E*. *huxleyi* strain behaves largely homeostatic [20]. Irrespective of the pCO_2_ acclimation, high light increased growth rates also in the haploid life-cycle stage, which is in line with previous observations [20,41,43].

### Effects on the composition of biomass

#### Effects of ocean acidification on the diploid life-cycle stage

**Fig 3:**
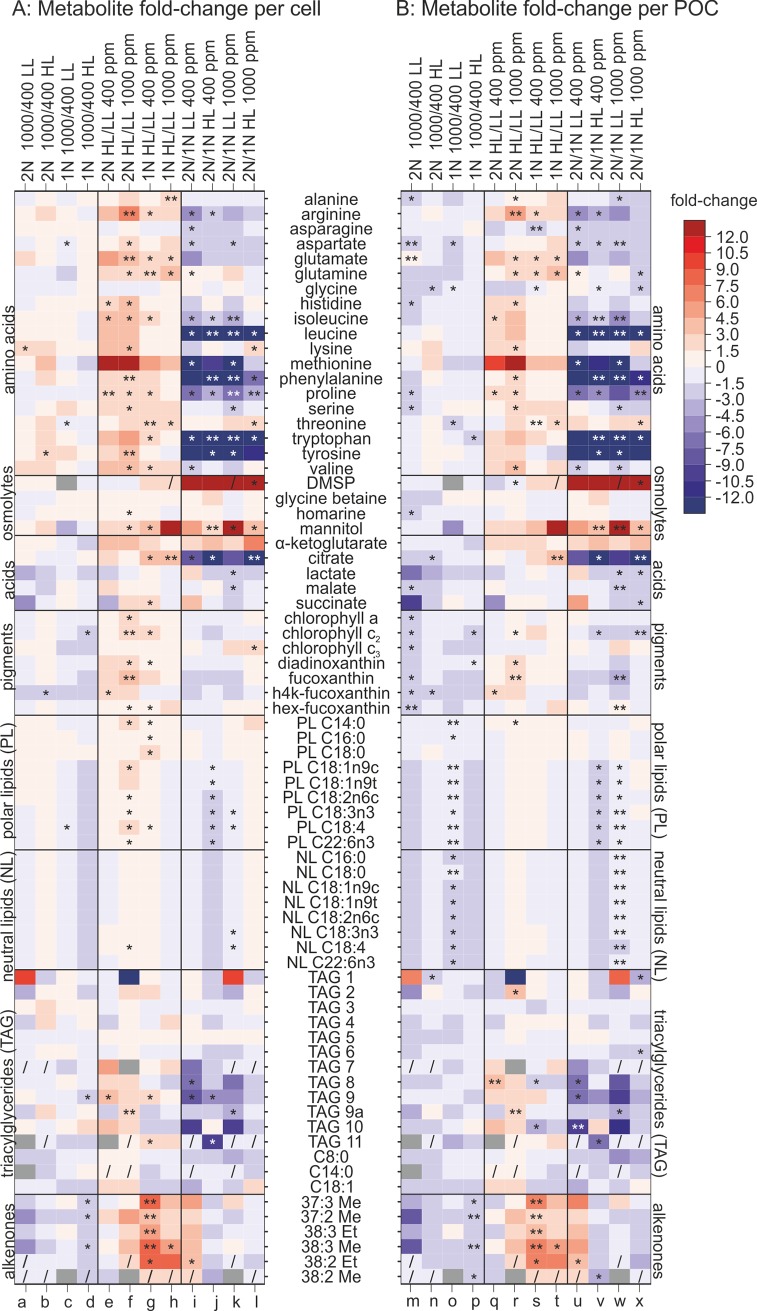
Heatmap of fold-changes of metabolites. Fold-changes of metabolites in different pCO_2_ (400 vs. 1000 μatm CO_2_) and light acclimations (LL vs. HL, i.e., 50 vs. 300 μmol photons m^-2^ s^-1^) for diploid (2N) and haploid (1N) *E*. *huxleyi* life-cycle stages. Fold-changes of the mean values of three biological replicates were calculated. Increases are indicated in red, decreases in blue, grey boxes indicate that the metabolite was not detected in the sample. Asterisks represent *p*-values as determined via Student’s *t*-test (* represents *p*-values ≤0.05, ** represents *p*-values ≤0.01). Slashes ‘/’ indicate manually added fold-change tendencies for metabolites where the fold-change could not be calculated due to metabolites being absent or lower than the detection limit in one of the treatments. A) Metabolite fold-change per cell, B) Metabolite fold-change per POC.

Overall, the effects of OA on the cellular metabolic composition of the diploid stage grown under high or low light intensities (Fig 3, columns a, b) were numerically small, most metabolites experiencing average fold-changes between 1.5 and 4.5-fold, irrespective of the direction of regulation. Under high light, OA caused most compounds to be slightly increased per cell, however significance could only be determined for the increase in tyrosine content (1.7-fold) and the decreased contents of 19’-hexanoyloxy-4-ketofucoxanthin (h4k-fucoxanthin; -1.7-fold). In the low light treatment, OA resulted in slightly increased cellular abundances for several amino acids, however only lysine content was significantly increased (2.2-fold).

Consequentially, this data does not indicate specific or systematic effects of OA on the cellular content of biochemicals, suggesting that OA does not cause specific deviations or imbalances in the metabolome, as was found for example for starvation scenarios in macronutrients like nitrogen and phosphorus [36,44], as well as for shifts in salinity [45].

However, irrespective of the overall small magnitude of responses, we observed slight but consistent increases in the per-cell contents of most amino acids and some osmolytes throughout the dataset. Since these metabolite quotas are expressed per cell, the slight increases may simply reflect increases in overall organic cell mass under OA, as often observed in this and some other strains of *E*. *huxleyi* [6,20,46]. We therefore re-normalized the metabolite quotas to the POC quotas (in pg POC cell^-1^) obtained from the earlier study by Rokitta and Rost [20], where POC increased in the OA and high light set-ups. Looking at the POC-normalized compound quotas (Fig 3, columns m-x), it becomes clear that most metabolites were in fact slightly *less* abundant per unit of biomass. This is remarkable for the detected pigments, because this is in line with the decreased POC-normalized content of chlorophyll a under OA as previously observed for this strain [20,46]. Viewing the data from these two different aspects, it must be concluded that OA increases the overall biomass per cell but does not cause significant alterations of its composition.

#### Effects of high light on the diploid life-cycle stage

High light intensity increased the abundance of many biochemicals in the diploid life-cycle stage (Fig 3, columns e, f), and the changes had an overall higher dynamic range than observed in response to OA. As a consequence, more than 30 out of 75 metabolites underwent significant changes due to high light, irrespective of the pCO_2_ levels. High light significantly increased the cellular contents of histidine (2.7-fold), isoleucine (3.9-fold) and proline (2.6-fold), as well as mannitol (2.1-fold), the pigment h4k-fucoxanthin (2.3-fold) and TAG 9 (3.1-fold) in the low pCO_2_ acclimations (Fig 3, column e). The effects of high light intensity were even more pronounced under OA, e.g., the cellular abundances of the amino acids arginine (6.2-fold), aspartate (1.8-fold), glutamate (3.4-fold), glutamine (2.3-fold), histidine (3.8-fold), isoleucine (4.4-fold), lysine (3.1-fold), phenylalanine (3.0-fold), serine (2.6-fold), proline (2.8-fold), tyrosine (3.6-fold) and valine (4.0-fold) were significantly increased (Fig 3, column f). The methionine contents appeared strongly increased in response to high light (14.4- to 35.1-fold), but these effects lacked statistical significance. Furthermore, the cellular abundances of all examined pigments increased under high light, an effect that was stronger under elevated pCO_2_. The changes were significant for chlorophyll a (1.5-fold), chlorophyll c_2_ (1.7-fold), 19’-hexanoyloxyfucoxanthin (hex-fucoxanthin) (2.1-fold) and also diadinoxanthin (2.1-fold). The acclimation to high light increased the cellular contents of lipids, especially when pCO_2_ was high, the changes being significant for most of the investigated polar lipids, i.e., C14:0 (1.7-fold), C18:1n9c (1.5-fold) and C18:4 (1.5-fold). Also triacylglycerides and alkenones showed increased cellular abundance in response to high light when pCO_2_ was high, as seen especially for TAG 9a (2.2-fold).

With respect to the metabolite composition and contents, the applied high light intensity is a much stronger driver than the applied OA scenario. The cell-normalized contents (Fig 3, columns e, f) of most amino acids experienced notable increases under high light, as did the small carboxylic acids, pigments and the lipid compounds, here especially the alkenones. However, many of these increases in cellular metabolite contents can be attributed simply to the higher cellular biomass per cell that is typically caused by high light. After re-normalizing the metabolite abundances to biomass itself (Fig 3, columns q, r), it becomes clear that the contents of carboxylic acids and light-harvesting pigments actually decrease in the biomass, as has been suggested before by transcriptomic assessments [15,20]. The biomass-normalized metabolite patterns of most amino acids, photo-protective pigments and some TAGs are largely preserved, suggesting small but real shifts in biomass-composition in response to high-light acclimation. These involve higher relative contents of most analysed amino acids, possibly, because the light-driven N-assimilation is more intense under high light. Also the increased content of the sugar-alcohol mannitol that functions as a storage compound [47] indicates the higher need to deposit photosynthate when exposed to high irradiances. Furthermore, the carotenoid accessory pigment fucoxanthin, and its derivatives, as well as the photoprotective pigment diadinoxanthin, which is involved in the xanthophyll-cycle [48,49], were present in higher abundances in the biomass of high-light grown diploid cells, likely to assure functionality of photosynthesis in this condition and to enhance non-photochemical quenching to dissipate light stress.

#### Effects of ocean acidification on the haploid life-cycle stage

The biochemical composition of the haploid life-cycle stage was mostly non-responsive to OA (Fig 3, columns c, d), and only eight out of 75 examined metabolites showed significantly altered cellular abundances across the two applied light levels. In the high-light acclimated haploid cells, OA did not notably affect the cellular contents of amino acids (Fig 3 column c). Like in the low-light acclimation, OA caused slight decreases in the abundances of all examined pigments in high-light acclimated cells, the decrease in chlorophyll c_2_ being significant (-1.7-fold). All fatty acids deriving from polar and non-polar lipids, except one, generally appeared to decrease in abundance, although data is lacking statistical significance. While no clear effects could be seen in the cellular contents of TAGs, OA clearly decreased abundances of all examined alkenones in haploid cells grown under high light.

In the low-light acclimated haploid cells, the cellular contents of most amino acids were also only insignificantly affected by OA. No general trend could be observed and only aspartate (-1.4-fold) and threonine (-1.4-fold) were significantly decreased. The abundances of all examined photosynthetic pigments slightly decreased in response to OA in the low-light treatment, although statistical significance is lacking. Most of the fatty acids derived from polar and non-polar lipids significantly decreased in abundance (Fig 3, column d).

The homeostatic behavior of this strain is well supported by the cell-normalized metabolite patterns observed here (Fig 3, columns c, d), which show even less significant changes in response to OA than the diploid stage, as no metabolite changed more than 3-fold. Re-normalization to POC (Fig 3, columns o, p) does not show many alterations either, underlining the previously postulated homeostatic behavior of the haploid life-cycle stage [20].

#### Effects of high light on the haploid life-cycle stage

In cells of the haploid life-cycle stage that were grown under low pCO_2_, high light intensity increased the abundances of most amino acids and all of the examined small carboxylic acids (Fig 3, column g). Significant changes were detected for arginine (2.5-fold), glutamate (2.0-fold), glutamine (2.2-fold), isoleucine (1.7-fold), proline (1.6-fold), threonine (1.8-fold), tryptophan (1.9-fold) and valine (1.8-fold), as well as citrate (3.1-fold), succinate (1.6-fold) and mannitol (4.2-fold). Most examined pigments were slightly increased in response to high light, significance was determined for chlorophyll c_2_ (2.1-fold) and diadinoxanthin (1.4-fold). All examined fatty acids were generally increased in abundance in response to high light, with significant changes in the cellular contents of the polar lipids C14:0 (1.5-fold), C16:0 (1.5-fold), C18:0 (1.6-fold) and C18:4 (1.5-fold). Alkenones significantly increased in cellular abundance, with fold-changes ranging between 1.5- and 8-fold.

Also in haploid cells grown under OA (Fig 3, column h), high light intensity increased cellular abundances of most amino acids, and significant changes were detected for alanine (1.7-fold), glutamine (4.4-fold), glutamate (2.4-fold) and threonine (2.5-fold). Among the small carboxylic acids, only citrate was significantly increased (3.5-fold). Regarding pigments, high light slightly decreased abundances in cells grown under high pCO_2_, but none of these small changes was significant. Similarly, high light did not significantly affect the contents of fatty acids derived from polar and non-polar lipids. In contrast, high light increased alkenone contents of cells acclimated to high pCO_2_, with significant effects being determined for the alkenone C38:3 Me (5.9-fold; Fig 3, column h).

Like in the diploid life-cycle stage, the metabolome of the haploid stage changed more intensively under the applied higher irradiance than under the applied OA treatment, showing increased per-cell contents of various amino acids, osmolytes, pigments and many alkenones. The re-normalization with literature-derived POC data [20], confirmed that these signals indeed derive from increased metabolite contents per biomass and are not an artifact derived from overall higher biomass per cell (Fig 3, columns s, t).

#### Comparison between the ploidy stages

The comparison of compound quotas between the two different life-cycle stages (Fig 3, columns i-l) yielded the highest dynamic range of data, with metabolite fold-changes ranging from -130 for methionine to 17300 for DMSP, indicating fundamental differences between the two cell types. Since these differences were an inherent property of both life-cycle stages, they were largely independent from the accompanying light intensities and/or pCO_2_ acclimations and thus recognizable across all four acclimation settings (Fig 3 columns i-l). Most amino acids had lower cellular abundances in the diploid life-cycle stage, especially arginine (-2.8 to -4.8-fold), aspartate (-1.7-fold), isoleucine (-1.6 to -4.1-fold), leucine (-29.6 to -129.1-fold), methionine (-17.5 to -31.6-fold) and proline (-3.4 to -6.8-fold), as well as the aromatic amino acids phenylalanine (-7.4 to -18.5-fold), tryptophan (-20.5 to -87.6-fold) and tyrosine (-19.3 to -60.8-fold). The diploid stage contained considerably less citrate (-14.7 to -23.2-fold), unsaturated polar lipids (on average -1.5-fold) as well as TAG 9 (-4.7 to -7.5-fold), whereas mannitol (2.4 to 27.0-fold) was more abundant in the diploid stage. The diploid life-cycle stage contained vastly more dimethylsulphopropionate (DMSP; 460- to 17300-fold) than the haploid stage, in which this osmolytic compound was either very low or not detectable at all (which is the reason for the fraction number to become very large easily). No data is available for the fold-change of DMSP in the comparisons of the haploid life-cycle stage grown under low light and high pCO_2_ (Fig 3, column c, h and k), because the DMSP content of this sample was below the detection limit. In the corresponding diploid grown under low light and high pCO_2_, the highest DMSP concentration of all samples was determined, the high contrast between the stages is shown visually (Fig 3, column k) but can’t be given in numbers. Diploid *E*. *huxleyi* cells are known to be prominent producers of DMSP in the marine system. As shown previously [36,50], the haploid stage did not contain significant amounts of this osmolyte, but higher amounts of methionine (up to 30 fold), a precursor of DMSP [51,52]. It seems that the excess of the DMSP lyase enzyme in the haploid stage [10,53] is responsible for both, absence of DMSP as well as the accumulation of the precursor, which seems to not be consumed by other significant processes.

The comparison of the two life-cycle stages (Fig 3, columns i-l, u-x) indicated large differences between the cell types that persist irrespective of light and pCO_2_ acclimation. These differences were also not influenced by a re-normalization to cells or biomass, because the metabolite contents diverged so strongly that the comparably small numerical differences in POC quota could not alter the overarching patterns. Most free amino acids had notably lower abundances in the diploid life-cycle stage, especially the aromatic amino acids phenylalanine (ranging from -7.44 to -18.47), tryptophan (from -20.53 to -87.61) and tyrosine (from -19.26 to -60.77). The high contents of aromatic amino acids in the haploid stage might suggest a higher activity of the shikimate pathway that also produces precursors for, e.g., mycosporine-like amino acids [54]. These compounds function as antioxidants and protection against UV stress [55] and a higher content in the haploid stage might thus be an example of life-cycle stage-specific niche occupation [10]. Many of the small carboxylic acids, especially citrate, are involved in primary respiratory- as well as lipid metabolism. The higher reliance of the haploid stage on these parts of metabolism was postulated from transcriptome analyses before [10] and might thus explain higher abundances of citrate. The provision of respiratory energy for the flagellar movement seems to require high carbon turnover and increased levels of mitochondrial oxidation [36,56] although calcification also has high energetic costs [57].

Surprisingly, the haploid stage increased its alkenone content relatively stronger than the diploid stage in response to high light intensity. These results are in line with the observed increase of alkenones exclusively in the haploid stage under nitrogen starvation [36], further confirming partially different properties of the life-cycle stages with regard to susceptibility to external stress and storage molecule buildup. Slight increases in diadinoxanthin and h4k-fucoxanthin under high light indicate the same protective rearrangements in the photosynthetic apparatus that were observed for the diploid stage. At the same time, many TAGs were slightly downregulated in the haploid life-cycle stage, suggesting slight shifts from TAGs to alkenones as storage lipids under high light intensities. Apparently, high light induces relatively stronger changes in the haploid stages main storage components like mannitol and alkenones. With respect to absolute contents, due to coccolith production, the diplont is still a stronger producer [57].

### Comparison with previously published data

We used the same microalgal strains, cultivation techniques and methods as previously presented in Wördenweber et al. [36]. Differences existed only in the cultivation medium and the temperature (enriched seawater and 15°C in this study vs. artificial seawater and 20°C in Wördenweber et al. [36]). The parallelisms between this and the mentioned study allowed for an intercomparison of the life-cycle stages (grown under nutrient replete conditions, high light intensities and contemporary pCO_2_) that showed some differences as well as similarities. We observed partially large differences in metabolite concentrations between the present experiment and Wördenweber et al. [36], with the directions of change not being consistent for all metabolites. For instance, most amino acids were in this experiment less abundant in the diploid life-cycle stage, whereas in Wördenweber et al. [36], about half of the amino acids were more abundant in the diploid life-cycle stage. However, most amino acids that were prominently abundant in the haploid stage in this experiment, behaved alike in the previous experiment (except phenylalanine and methionine).

The diploid life-cycle stage reproducibly contained vastly more DMSP than the haploid stage, although the specific fold-changes vary greatly between this study and Wördenweber et al. [36]. This is because of the minimal amounts of DMSP detected in the haplont that also cause the fraction number to become very large easily.

The higher abundance of mannitol in the diploid stage as well as the higher abundance of citrate in the haploid stage were closely reproduced between the two studies and also most other small carboxylic acids were detected to have similar distributions. Also the stage-specific distributions of pigments and alkenones were alike in both experiments. In contrast, the polar and neutral lipid concentrations as well as the contents of TAGs differ between the studies for almost all detected compounds, indicating that the cellular composition of lipids is subject to great biological variability. In contrast to Wördenweber et al. [36], we found generally lower amounts of lipid compounds in the diplont than in the haplont.

Mausz and Pohnert [56] also compared the metabolome of the same diploid and haploid strains. Although their approach and applied methods were different, there is an overlap in the metabolites identified. In line with this study, they also found higher isoleucine and citrate contents in the haplont and higher mannitol content in the diplont.

## Conclusions

The obtained biochemical data show that OA, while significantly affecting the transcriptomes of haploid and diploid life-cycle stages [19], has far smaller effects on the metabolic phenotypes. Even though we have in our analytical approach intentionally modified the original ‘*in-vivo* metabolome’, the analyses of biochemical compounds after treatment, e.g. hydrolysis of (un)polar lipids, suggest that OA has a negligible effect on the overall composition of biomass and does not alter the contribution of lipophilic compounds. Based on our data, no statements on the abundance of glucans can be made, but the development of analytical protocols for this diverse and somewhat cryptic class of biopolymers should be a priority for future studies. Whereas profound emergencies like nutrient starvation severely affect the physiology of the cells [15,36], high pCO_2_ and concomitant acidification seem to only slightly modulate the steady state fluxes between the assessed metabolite pools. The rearrangements in the biochemical pathways of plastidary and lipid metabolism that were previously postulated based on transcriptomics were often indicated, but their order of magnitude was either too small to be resolvable or masked by the comparably large biological variance, underlining the flexibility of cellular biochemical networks. Some trends deduced from the transcriptome studies could be reproduced, namely overall increased biomass production and shifts in pigmentation [19]. The applied light treatment was, however, shown to be a much stronger driver of the metabolome than OA, increasing cellular N-assimilation as well as carbon and lipid storage. In this respect, it may be deduced that OA, if it does not explicitly harm exquisite cell-physiological processes like calcification, or poses severe energetic constraints that feed forward to growth rates, does not seem not constitute a problem for microalgal photosynthesis and biomass buildup.

## Supporting information

S1 Table Carbonate system. (XLSX)

S2 Table Alkenones. Alkenone-derived temperature signals.
(XLSX)

S3 Table Growth and size. (XLSX)

S4 Table Supplementary data. Concentrations and fold-changes of metabolites.
(XLSX)
